# Presepsin is a more useful predictor of septic AKI and ARDS for very-old sepsis patients than for young sepsis patients in ICUs: a pilot study

**DOI:** 10.1186/s13104-024-06719-6

**Published:** 2024-02-20

**Authors:** Yuichiro Shimoyama, Noriko Kadono, Osamu Umegaki

**Affiliations:** Department of Anesthesiology, Intensive Care Unit, Osaka Medical and Pharmaceutical University, Osaka Medical and Pharmaceutical University Hospital, 2-7 Daigaku-Machi, Takatsuki, Osaka 569-8686 Japan

**Keywords:** Presepsin, Sepsis, Septic acute kidney injury, Acute respiratory distress syndrome, Very-old sepsis patients

## Abstract

**Objective:**

Sepsis is a syndrome of life-threatening organ dysfunction. This study aimed to determine whether presepsin is a useful predictor of septic acute kidney injury (AKI), acute respiratory distress syndrome (ARDS), disseminated intravascular coagulation (DIC), and shock in very-old sepsis patients aged 75 years in intensive care units (ICUs).

**Results:**

A total of 83 adult patients diagnosed with sepsis were prospectively examined and divided into two groups: those aged 75 years and older (over 75 group) and those aged younger than 75 years (under 75 group). Presepsin values were measured after ICU admission. Inflammation-based prognostic scores were also examined. For category classification, total scores (“inflammation-presepsin scores [iPS]”) were calculated. Presepsin values, inflammation-based prognostic scores, and iPS were compared between patients with septic AKI, ARDS, DIC, or shock and those without these disorders in the over 75 and under 75 groups. Areas under the curve of presepsin for predicting septic AKI and ARDS in the over 75 group were both > 0.7, which were significantly higher than those in the under 75 group. In conclusion, presepsin is a more useful predictor of septic AKI and ARDS for very-old sepsis patients (over 75 years) than for younger sepsis patients (under 75 years).

**Supplementary Information:**

The online version contains supplementary material available at 10.1186/s13104-024-06719-6.

## Introduction

Sepsis, a syndrome of life-threatening organ dysfunction [1, 2], remains a critical condition associated with high mortality in critically ill patients [3]. The early diagnosis of severe sepsis and early initiation of appropriate therapy are crucial [4]. Procalcitonin (PCT) is the most frequently used diagnostic marker for sepsis. However, PCT values can be falsely elevated without bacterial infection due to other conditions such as invasive surgery and critical burn injuries [5–7].

Presepsin, a subtype of N-terminal fragment of soluble CD14 (CD14-ST), is a 13-kDa protein [8] released into the circulation during systemic infections secondary to monocyte activation [9]. It is a novel diagnostic biomarker for bacterial sepsis and is also useful for predicting prognosis and monitoring the severity of sepsis [10, 11]. The diagnostic and prognostic abilities of presepsin have been reported to be higher than those of PCT and C-reactive protein (CRP) [12, 13]. Presepsin is released early (i.e., within 2 h) after the onset of sepsis and can be measured in less than 17 min by chemiluminescent enzyme immunoassay (CLEIA) using a compact, fully automated immunoanalyzer (PATHFAST; Mitsubishi Chemical Medience Corporation, Tokyo, Japan) [14, 15].

The incidence rates of sepsis and related complications are much higher in very-old patients than in younger patients [16, 17]. Inflammation-based prognostic scores, including the Glasgow Prognostic Score (GPS; based on serum CRP and albumin levels), neutrophil to lymphocyte ratio (NLR), platelet to lymphocyte ratio (PLR), Prognostic Nutritional Index (PNI; based on albumin and lymphocyte counts), and Prognostic Index (PI; based on serum CRP and white blood cell counts), are well-known prognostic biomarkers for several types of cancer [18]. Accordingly, the present study aimed to test the following hypothesis: presepsin is a more useful predictor of septic acute kidney injury (AKI), acute respiratory distress syndrome (ARDS), disseminated intravascular coagulation (DIC), and shock for very-old sepsis patients aged 75 years and older than for those aged younger than 75 years in ICUs.

## Main text

### Methods

#### Patients and study design

**Fig. 1:**
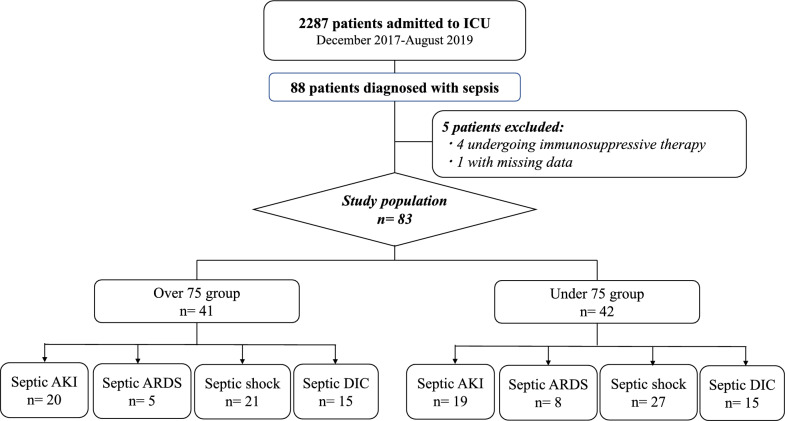
Flowchart for study population selection. A total of 83 adult patients aged 18 years and older who were diagnosed with sepsis according to the Sepsis-3 definition and admitted to the ICU were prospectively examined from December 2017 to August 2019. Patients were divided into two groups: those aged 75 years and older (over 75 group) and those aged younger than 75 years (under 75 group)

The present study adopted the study design, informed consent procedure, patient inclusion and exclusion criteria, and the definition of “inflammation-presepsin scores [iPS]” previously described [19, 20]. Patients were divided into the “over 75 group,” comprising those aged 75 years and older, and the “under 75 group,” comprising those aged younger than 75 years (Fig. 1). Septic AKI referred to stage 1 kidney disease as defined by the Kidney Disease: Improving Global Outcomes (KDIGO) classification [21]. For septic ARDS, DIC, and shock, the Berlin definition [22], the Japanese Association for Acute Medicine DIC diagnostic criteria [23], and the Sepsis-3 definition [1], were used, respectively. Presepsin values measured using CLEIA, which takes less than 17 min to obtain results [15], inflammation-based prognostic scores, iPS, and changes (⊿) in presepsin values relative to baseline values at each sampling point were compared between patients with septic AKI, ARDS, DIC, or shock after ICU admission and those without these complications in the over 75 and under 75 groups.

#### Statistical analysis

Categorical data are presented as percentages, and continuous data are presented as medians with interquartile ranges. Fisher’s exact test and the Mann–Whitney U test were used to analyze categorical data and continuous data, respectively. Areas under the curve (AUCs) of presepsin, inflammation-based prognostic scores, iPS, and ⊿presepsin, cut-off values, sensitivities, and specificities were calculated. AUCs of presepsin were compared between the over 75 and under 75 groups using the Mann–Whitney U test. Multivariate logistic regression analysis was performed to determine whether presepsin values, inflammation-based prognostic scores, iPS, ⊿presepsin, SOFA, and quick SOFA (qSOFA) (with P < 0.05 in univariate analysis) were predictors of septic AKI, ARDS, DIC, and shock. In addition, ROC curve analysis and the log-rank test were performed to compare the prognostic ability of these variables. P < 0.05 was considered statistically significant. All statistical analyses were performed using JMP software version 11.0.0 (SAS Institute Inc, NC).

## Results

Patient baseline characteristics are shown in Table 1 (data were sourced from our previous publication: https://bmcnephrol.biomedcentral.com/articles/0.1186/s12882-021–02422-x) [20]. No significant differences were observed in age, sex, and underlying conditions among patients with septic AKI, ARDS, DIC, and shock (Table 2). ROC curve analyses revealed that the AUCs of presepsin for predicting septic AKI and ARDS in the over 75 group were both significantly higher than those in the under 75 group, at > 0.7 (Additional file 1: Table S1 and Additional file 2: Table S2). The multivariate logistic regression analysis of presepsin on Day 1 and iPS-PNI (P < 0.05 in univariate analysis, Table 2) revealed that iPS-PNI was a predictor of septic AKI in the under 75 group (Additional file 3: Table S3). Furthermore, ROC curve analyses revealed that iPS-PNI had higher specificity for predicting mortality than presepsin on Day 1 in the under 75 group (Additional file 4: Table S4). The log-rank test revealed that only iPS-PNI was a significant predictor of mortality in the under 75 group (Additional file 5: Table S5).

**Table 1:** Patient baseline characteristics

Variable	n = 83	
Age (years)	74.0	(65.5–78.5)
Sex (male) (%)	51.0	(61.4)
Cancer (%)	40.0	(48.2)
Coronary artery disease (%)	4.0	(4.8)
Diabetes mellitus (%)	10.0	(12.0)
Hypertension (%)	21.0	(25.3)
Albumin (g/dL)	2.3	(1.8–3.0)
CRP (mg/dL)	10.4	(3.7–17.5)
WBC (× 10^9^ l^−1^)	10.9	(5.4–15.4)
Neutrophil count (× 10^9^ l^−1^)	8.7	(3.56–13.29)
Lymphocyte count (× 10^9^ l^−1^)	0.5	(0.299–0.927)
Platelet count (× 10^4^ mm^−3^)	17.8	(11.5–26.5)
Fibrinogen (mg/dL)	609.0	(378–711)
Survival (dead) (%)	26.0	(31.3)
AKI (%)	38.0	(45.8)
ARDS (%)	13.0	(15.7)
Shock (%)	48.0	(57.8)
DIC (%)	30.0	(36.1)
Presepsin on Day 1 (pg/mL)	1051.5	(569–1819.3)
Presepsin on Day 2 (ng/mL)	1016.5	(538–2156)
Presepsin on Day 3 (ng/mL)	802.0	(480.5–1825)
Presepsin on Day 5 (ng/mL)	1043.0	(480–1616)
ΔPresepsin Day 2—Day 1 (pg/mL)	− 21.50	(-246.5–274.75)
ΔPresepsin Day 3—Day 1 (pg/mL)	− 38.50	(-748.5–304)
ΔPresepsin Day 5—Day 1 (pg/mL)	− 59.50	(-745.75–635.5)
GPS	1.0	(1–2)
NLR	12.6	(4.53–26.35)
PLR	321.9	(195.63–543.69)
PI	1.0	(1–2)
PNI	26.6	(21.26–33.72)
SOFA	8.0	(5–11)
qSOFA	2.0	(1–3)

*CRP* C-reactive protein, *WBC* white blood cell, *AKI* acute kidney injury, *ARDS* acute respiratory distress syndrome, *DIC* disseminated intravascular coagulation, *GPS* Glasgow Prognostic Score, *NLR* neutrophil to lymphocyte ratio, *PLR* platelet to lymphocyte ratio, *PI* Prognostic Index; *PNI* Prognostic Nutritional Index, *SOFA* Sequential Organ Failure Assessment; qSOFA, quick SOFA The source of data in Table 1 is our previous publication below (https://bmcnephrol.biomedcentral.com/articles/10.1186/s12882-021-02422-x) [20]

**Table 2:** Predictors of septic AKI, ARDS, shock, and DIC (univariate analysis)

Variable	Over 75 group				Under 75 group			
	AKI (n = 20)	ARDS (n = 5)	Shock (n = 21)	DIC (n = 15)	AKI (n = 19)	ARDS (n = 8)	Shock (n = 27)	DIC (n = 15)
	P value	P value	P value	P value	P value	P value	P value	P value
Age	0.150	0.835	0.7622	0.966	0.9899	0.5211	0.180	0.813
Sex	0.7411	0.6404	0.748	0.310	1.0000	1.0000	1.000	0.511
Cancer	0.7512	1.0000	1.000	0.104	0.7555	0.4307	0.754	0.532
Coronary artery disease	0.4872	1.0000	0.475	1.000	0.581	0.4787	1.000	1.000
Diabetes mellitus	0.2308	1.0000	0.596	1.000	0.034	0.6012	0.686	0.225
Hypertension	0.0648	0.3059	0.281	0.457	0.180	0.1743	0.717	1.000
Albumin	0.3603	0.0714	0.674	0.823	0.9495	0.5422	0.608	0.554
CRP	0.0212	0.1707	0.490	0.175	0.1086	0.8476	0.096	0.106
WBC	0.8221	0.5809	0.424	0.922	0.1113	0.4420	0.007	0.014
Neutrophil	0.5365	0.6381	0.675	0.727	0.2608	0.3698	0.003	0.081
Lymphocytes	0.8003	0.2607	0.010	0.342	0.4560	0.3207	0.045	0.013
Platelet count	0.1600	0.8061	0.012	0.001	0.2156	0.4232	0.030	0.000
Fibrinogen	0.6510	0.2946	0.019	0.101	0.4808	0.6076	0.041	0.113
Survival	0.0958	0.3446	0.527	0.502	0.143	0.0115	0.286	1.000
AKI		0.1723	0.527	0.008		0.0565	0.207	0.049
ARDS	0.3416		0.654	0.345	0.112		1.000	0.425
Shock	0.3431	0.6544		0.001	0.108	0.6888		0.180
DIC	0.0079	0.3446	0.001		0.055	0.4255	0.180	
Presepsin on Day 1	0.023	0.011	0.766	0.043	0.0083	0.0564	0.725	0.425
Presepsin on Day 2	0.045	0.007	0.537	0.040	0.0500	0.0336	0.542	0.087
Presepsin on Day 3	0.0527	0.022	0.598	0.065	0.3111	0.1741	0.586	0.890
Presepsin on Day 5	0.0433	0.202	0.380	0.558	0.7728	0.5713	0.089	0.435
ΔPresepsin Day 2—Day 1	0.6434	0.779	0.111	0.171	0.4526	0.2937	0.220	0.058
ΔPresepsin Day 3—Day 1	0.9738	0.865	0.495	0.947	0.1734	0.4995	0.092	0.612
ΔPresepsin Day 5—Day 1	0.9292	0.111	0.684	0.110	0.3683	0.4669	0.146	0.588
GPS	0.268	0.109	0.520	0.525	0.355	0.612	0.247	0.140
NLR	0.938	0.158	0.261	0.977	0.958	0.844	0.074	0.783
PLR	0.220	0.359	1.000	0.009	0.067	0.818	0.588	0.379
PI	0.656	0.444	0.933	0.954	0.136	0.912	0.025	0.878
PNI	0.335	0.140	0.657	0.645	0.875	0.278	0.534	0.296
iPS-GPS	0.019	0.042	0.552	0.051	0.237	0.313	0.876	0.133
iPS-NLR	0.043	0.735	0.288	0.075	0.153	0.126	0.250	0.916
iPS-PLR	0.237	0.419	0.839	0.786	0.546	0.098	0.233	0.575
iPS-PI	0.007	0.225	0.348	0.039	0.020	0.568	0.675	0.749
iPS-PNI	0.211	0.301	0.102	0.432	0.006	0.706	0.548	0.721
SOFA	0.051	0.347	0.142	0.078	0.238	0.113	0.069	0.440
qSOFA	0.633	0.692	0.966	0.603	0.091	0.533	0.370	0.613

*AKI* acute kidney injury, *ARDS* acute respiratory distress syndrome, *DIC* disseminated intravascular coagulation, *CRP* C-reactive protein, *WBC* white blood cell; *GPS* Glasgow Prognostic Score, *NLR* neutrophil to lymphocyte ratio, *PLR* platelet to lymphocyte ratio, *PI* Prognostic Index, *PNI* Prognostic Nutritional Index;

*iPS* inflammation-presepsin score, *SOFA* Sequential Organ Failure Assessment, *qSOFA* quick SOFA

## Discussion

In the present study, ROC curve analyses revealed that the AUCs of presepsin for predicting septic AKI and ARDS in the over 75 group were both > 0.7, which were significantly higher than those in the under 75 group (Tables S1 and S2). These findings suggest that presepsin is a more useful predictor of septic AKI and ARDS for very-old sepsis patients than for younger sepsis patients. Presepsin values on Days 1–3 (AKI) and Days 1–2 (ARDS) had a higher sensitivity for predicting septic AKI and ARDS in the over 75 group (Additional file 1: Table S1), suggesting that presepsin values may offer an easy “rule out” test for predicting AKI and ARDS in the over 75 group at early stages after ICU admission. Compared with younger patients, very-old patients generally have depressed immune system and white blood cell functions, which may alter their response to infectious stimuli. Due to aging, hematopoietic stem cells differentiate less into common lymphoid progenitors and more into common myeloid progenitors over time, and this shift results in decreased differentiation into lymphoid cells (T cells, B cells) and increased differentiation into myeloid cells (granulocytes, monocytes) [24, 25]. Since presepsin is secreted from granulocytes, aging may have an effect on presepsin secretion.

In the present study, cut-off values of presepsin for predicting septic AKI and ARDS in the over 75 group (Additional file 1: Table S1) were higher than those previously reported for diagnosing sepsis (300–500 pg/mL) and severe sepsis (500–1,000 pg/mL) [26, 27]. Moreover, cut-off values of presepsin were higher for predicting septic ARDS than for predicting septic AKI at all sampling points in the over 75 group (Additional file 1: Table S1). These findings suggest the need to adopt higher age-specific presepsin cut-off values for predicting septic AKI and ARDS in very-old sepsis patients. However, given the considerable overlap of presepsin cut-off values for predicting each complication associated with sepsis, it may not be feasible to use presepsin values alone as a definitive index for diagnosing the onset of complications. Hence, in addition to using these cut-off values, clinical findings of each patient must be comprehensively evaluated to make a diagnosis based on all information available.

We previously reported that NLR is superior to other inflammation-based prognostic scores in predicting mortality in patients with gastrointestinal perforation and pneumonia [28, 29]. The multivariate logistic regression analysis of presepsin on Day 1 and iPS-PNI (with P < 0.05 in univariate analysis, Table 2) revealed that iPS-PNI, but not presepsin on Day 1, was a predictor of septic AKI (Additional file 3: Table S3) in the under 75 group. Furthermore, ROC curve analyses revealed that iPS-PNI had higher specificity for predicting mortality than presepsin on Day 1 in the under 75 group (Additional file 4: Table S4). Thus, iPS-PNI may serve as an easy “rule in” test for predicting mortality in the under 75 group. The log-rank test revealed that only iPS-PNI was a significant predictor of 28-day (p = 0.017), 60-day (p = 0.039), 90-day (p = 0.039) and 180-day (p = 0.009) mortality (Additional file 5: Table S5), suggesting that iPS-PNI is a more useful predictor of mortality than presepsin in the under 75 group.

The AUC, sensitivity, and specificity of iPS-PNI for predicting septic AKI were 0.71, 89%, and 41%, respectively, showing a higher sensitivity than that of presepsin alone at baseline in the under 75 group (Additional file 1: Table S1). Thus, PNI combined with presepsin values may be useful as an easy “rule out” test at the time of ICU admission for predicting septic AKI in the under 75 group. The advantage of combining biomarkers to improve the predictive ability for severe sepsis and septic shock has been demonstrated in a previous study [30]. The AUC of presepsin values combined with Mortality in Emergency Department Sepsis (MEDS) score or Acute Physiology and Chronic Health Evaluation (APACHE) II score was significantly greater than the AUC of MEDS score or APACHE II score alone for predicting severe sepsis or septic shock [30]. We previously reported that the specificity for predicting septic ARDS was improved by combining presepsin values and GPS relative to using baseline presepsin values alone [19]. Pinato et al. reported the advantage of using PNI for predicting poor overall survival in patients with hepatocellular carcinoma [31]. Albumin replacement did not improve survival at 28 and 90 days in patients with severe sepsis and septic shock in the Albumin Italian Outcome Sepsis trial [32]. However, our results suggest that, among the many variables that can be examined for assessing inflammation, hypoalbuminemia and lymphocytopenia (albumin and lymphocyte counts used to calculate PNI) are crucial variables [20] for predicting septic AKI in the under 75 group. Serum albumin values are significantly correlated with presepsin values [33]. Zahorec et al. reported a correlation between the severity of the clinical course and extent of lymphocytopenia in oncological ICU patients following major surgery, sepsis, and septic shock [34].

No previous study has assessed the association between presepsin values, alone or together with inflammation-based prognostic scores, and septic AKI, ARDS, DIC and shock in very-old ICU patients aged 75 years and older with sepsis. Further studies will be needed.

## Conclusions

Presepsin is a more useful predictor of septic AKI and ARDS for very-old sepsis patients than for younger sepsis patients. On the other hand, iPS-PNI is a superior predictor of septic AKI and mortality compared with presepsin alone in patients under 75 years of age.

## Limitations

First, the present study was conducted at a single center. Second, we used a single biomarker, with no comparisons performed for other biomarkers. Third, presepsin values increase with declining renal function [33]. Hence, the diagnostic accuracy of presepsin values may be influenced by kidney function.

### Supplementary Information

**Additional file 1: Table S1.** Receiver operating characteristic curve analysis.

**Additional file 2: Table S2.** Comparison of AUCs of presepsin values (over 75 group vs under 75 group).

**Additional file 3: Table S3.** Predictors of septic AKI in the under 75 group (multivariate analysis).

**Additional file 4: Table S4.** Receiver operating characteristic curve analysis on prognosis (under 75 group).

**Additional file 5: Table S5.** Log-rank test (under 75 group).

## Data Availability

The datasets used and/or analyzed during the current study are available from the corresponding author on reasonable request.

## Abbreviations

SOFA: Sequential Organ Failure Assessment
PCT: Procalcitonin
ICU: Intensive care unit
CRP: C-reactive protein
CLEIA: Chemiluminescent enzyme immunoassay
GPS: Glasgow Prognostic Score
NLR: Neutrophil to lymphocyte ratio
PLR: Platelet to lymphocyte ratio
PNI: Prognostic nutritional index
PI: Prognostic Index
AKI: Acute kidney injury
ARDS: Acute respiratory distress syndrome
DIC: Disseminated intravascular coagulation
iPS: Inflammation-presepsin scores
KDIGO: Kidney Disease: Improving Global Outcomes
ROC: Receiver operating characteristic
AUC: Area under the curve
qSOFA: Quick sequential organ failure assessment
OR: Odds ratio
CI: Confidence interval
MEDS: Mortality in Emergency Department Sepsis
APACHE: Acute Physiology and Chronic Health Evaluation
