# Supplementary material for: Presepsin is a more useful predictor of septic AKI and ARDS for very-old sepsis patients than for young sepsis patients in ICUs: a pilot study
Source: BMC Res Notes. 2024 Feb 20;17:53. doi: 10.1186/s13104-024-06719-6 (PMC10877906; doi:10.1186/s13104-024-06719-6)
Supplement: Supplementary file 1 [file 13104_2024_6719_MOESM1_ESM.docx]

Presepsin on Day 2 (pg/mL)	0.68
Presepsin on Day 3 (pg/mL)	0.52
Presepsin on Day 5 (pg/mL)	0.61
ΔPresepsin Day 2 - Day 1 (pg/mL)	0.71
ΔPresepsin Day 3 - Day 1 (pg/mL)	0.56
ΔPresepsin Day 5 - Day 1 (pg/mL)	0.58
GPS	0.62
NLR	0.53
PLR	0.58
PI	0.51
PNI	0.60
iPS-GPS	0.63
iPS-NLR	0.51
iPS-PLR	0.55
iPS-PI	0.53
iPS-PNI	0.53

AUC, area under the curve; AKI, acute kidney injury; GPS, Glas
platelet to lymphocyte ratio; PI, Prognostic Index; PNI, Prognosti
acute respiratory distress syndrome; DIC, disseminated intravascu

ARDS,
iPS, inflammation-Presepsin score; DIC, disseminated intravascu

CRP, C-reactive protein; WBC, white blood cell; AKI, acute kidne
acute respiratory distress syndrome; DIC, disseminated intravascu
GPS, Glasgow Prognostic Score; NLR, neutrophil to lymphocyte r
to lymphocyte ratio; PI, Prognostic Index; PNI, Prognostic Nutrit
SOFA, Sequential Organ Failure Assessment; qSOFA, quick Se
Failure Assessment

CRP, C-reactive protein; WBC, white blood cell; AKI, acute kidne
acute respiratory distress syndrome; DIC, disseminated intravascu
GPS, Glasgow Prognostic Score; NLR, neutrophil to lymphocyte r
to lymphocyte ratio; PI, Prognostic Index; PNI, Prognostic Nutrit
SOFA, Sequential Organ Failure Assessment; qSOFA, quick Se
Failure Assessment
