# Supplementary material for: Presepsin is a more useful predictor of septic AKI and ARDS for very-old sepsis patients than for young sepsis patients in ICUs: a pilot study
Source: BMC Res Notes. 2024 Feb 20;17:53. doi: 10.1186/s13104-024-06719-6 (PMC10877906; doi:10.1186/s13104-024-06719-6)
Supplement: Supplementary file 2 [file 13104_2024_6719_MOESM2_ESM.docx]

Table S2. Comparison of AUCs of presepsin values (over 75 group vs under 75 gro

	P value
AKI	0.043
ARDS	0.043
Shock	0.564
DIC	0.083
