# Supplementary material for: Presepsin is a more useful predictor of septic AKI and ARDS for very-old sepsis patients than for young sepsis patients in ICUs: a pilot study
Source: BMC Res Notes. 2024 Feb 20;17:53. doi: 10.1186/s13104-024-06719-6 (PMC10877906; doi:10.1186/s13104-024-06719-6)
Supplement: Supplementary file 3 [file 13104_2024_6719_MOESM3_ESM.docx]

Table S3. Predictors of septic AKI in the under 75 group (multivariate analysis)

Variable	Odds ratio	95% CI	
iPS-PNI	5.622	1.165	27.134
Presepsin on Day 1	1.000	1.000	1.001

AKI, acute kidney injury; CI, confidence interval; iPS, inflammation-presepsin scor
Nutritional Index
