# Supplementary material for: Presepsin is a more useful predictor of septic AKI and ARDS for very-old sepsis patients than for young sepsis patients in ICUs: a pilot study
Source: BMC Res Notes. 2024 Feb 20;17:53. doi: 10.1186/s13104-024-06719-6 (PMC10877906; doi:10.1186/s13104-024-06719-6)
Supplement: Supplementary file 4 [file 13104_2024_6719_MOESM4_ESM.docx]

Table S4. Receiver operating characteristic curve analysis on prognosis (under 75 group)

| Variable | AUC | Cut-off | P value | Sensitivity |
|---|---|---|---|---|
| 28-day mortality | | | | |
| iPS-PNI | 0.65 | 2.00 | 0.231 | 0.33 |
| presepsin on Day 1 | 0.80 | 1275.00 | 0.004 | 0.83 |
| 60-day mortality | | | | |
| iPS-PNI | 0.65 | 2.00 | 0.172 | 0.29 |
| presepsin on Day 1 | 0.71 | 1275.00 | 0.079 | 0.71 |
| 90-day mortality | | | | |
| iPS-PNI | 0.65 | 2.00 | 0.172 | 0.29 |
| presepsin on Day 1 | 0.71 | 1275.00 | 0.079 | 0.71 |
| 180-day mortality | | | | |
| iPS-PNI | 0.70 | 2.00 | 0.032 | 0.33 |
| presepsin on Day 1 | 0.75 | 1275.00 | 0.012 | 0.78 |
