# Supplementary material for: Presepsin is a more useful predictor of septic AKI and ARDS for very-old sepsis patients than for young sepsis patients in ICUs: a pilot study
Source: BMC Res Notes. 2024 Feb 20;17:53. doi: 10.1186/s13104-024-06719-6 (PMC10877906; doi:10.1186/s13104-024-06719-6)
Supplement: Supplementary file 5 [file 13104_2024_6719_MOESM5_ESM.docx]

Table S5. Log-rank test (under 75 group)

Variable
28-day mortality
iPS-PNI
presepsin on Day 1
60-day mortality
iPS-PNI
presepsin on Day 1
90-day mortality
iPS-PNI
presepsin on Day 1
180-day mortality
iPS-PNI
presepsin on Day 1
